# Python-controlled multimodal UV-VIS hyperspectral imaging system

**DOI:** 10.1117/1.JBO.31.9.096003

**Published:** 2026-09-15

**Authors:** Elizabeth A. Bullard, Erin M. Stout, Oscar R. Benavides, Anaya Bawiskar, Lakhvir Singh, Ngoc Nhu Vu, Samuel Mabbott, Alex J. Walsh

**Affiliations:** Texas A&M University, Department of Biomedical Engineering, College Station, Texas, United States

**Keywords:** hyperspectral imaging, autofluorescence, optical system, characterization, Python

## Abstract

**Significance:**

Traumatic and chronic wound healing is a complex process that often requires careful observation by healthcare professionals. Noninvasive optical technologies such as hyperspectral reflectance imaging and tissue autofluorescence imaging detect changes in optical properties related to physiological parameters such as tissue perfusion. Macroscopic hyperspectral systems with a large field of view to visualize tissue structure and perfusion are well suited for wound care.

**Aim:**

Here, a custom-built benchtop hyperspectral and autofluorescence imaging system (HySAF) is optimized to measure tissue optical properties. It operates using a custom Python script to allow precise control over acquisition parameters.

**Approach:**

HySAF was built using off-the-shelf components, and Python code was written to control primary devices. HySAF’s performance was evaluated by imaging oxygenated and deoxygenated blood samples, scattering induced by nanoparticles, scorpion autofluorescence, and rat wounds.

**Results:**

HySAF has a large field of view (FOV) with 176.7  μm pixel resolution and 10 nm spectral resolution from 420 to 730 nm. Changes in absorbance were observed during blood deoxygenation, nanoparticle presence in cells, and healing wound tissue in rats, while autofluorescence distinguished anatomical parts of the scorpion.

**Conclusion:**

HySAF is capable of detecting changes in sample fluorescence and absorbance across visible light with large FOV and could be implemented in clinical applications such as monitoring and assessing wound healing.

## 1 Introduction

Wound management is a tremendous burden on patients and healthcare systems worldwide.^1^^–^^5^ In the United States, ∼2% of the population is estimated to have a chronic wound at any time, cumulatively resulting in an economic burden that likely exceeds $50 billion annually.^1^^,^^6^^,^^7^ Chronic and severe acute wounds require clinical interventions and monitoring to guide care.^8^^,^^9^ However, monitoring of wounds remains a challenge, with clinical visual inspection having an accuracy of only 60%.^10^ Wound healing progresses through three complex phases: inflammation, proliferation, and tissue remodeling^11^ that may overlap and differ across a wound bed.^12^ Healing can be impaired at any stage due to different causes such as lack of perfusion, tissue hypoxia, a prolonged inflammatory state of the tissue that prevents new tissue growth, impaired collagen deposition and remodeling by fibroblasts, or an overabundance of bacteria. Therefore, diagnostic assessments to guide treatment are ubiquitous in most cases.^13^ Although different causes of impaired wound healing have specific treatments, there are limited noninvasive medical devices or prognostic assays capable of identifying wounds in need of early intervention and guiding appropriate treatment.

Hyperspectral imaging (HSI) is a noninvasive, label-free method that acquires spatial images as a function of wavelength, resulting in a 3D dataset from which an intensity spectrum can be extracted for every pixel in the image.^14^ The interaction of visible light (400 to 700 nm) with materials such as tissue results in diffuse reflected light, which can be directly measured and used to assess tissue optical properties, such as absorption. Hyperspectral reflectance imaging (HSRI) can detect tissue perfusion and quantify tissue oxygenation due to the different light absorption properties of oxy- and deoxyhemoglobin in blood.^15^^,^^16^ Because hypoxic tissue environments can impede tissue repair,^7^ HSRI has potential for noncontact wound monitoring. Indeed, HSRI has been evaluated for detecting tumor margins, disease diagnosis, and wound monitoring.^17^^–^^19^ For wounds, HSRI allows estimation of wound size^20^ and quantification of oxygen saturation, hemoglobin content, and tissue water index in human wounds.^21^ Additional modalities such as fluorescence, confocal microscopy, or NIR spectroscopy can be combined with HRSI to provide additional structural or functional information about the imaged tissue.^22^^,^^23^

Autofluorescence, or fluorescence emitted by endogenous fluorophores, has potential for noninvasive *in vivo* monitoring of tissue. Both extracellular matrix proteins such as collagen and elastin, and intracellular metabolic coenzymes are fluorescent. Thus, the autofluorescence signature of a wound may reveal collagen remodeling and tissue inflammatory states. However, the spectral overlap of autofluorescence molecules within tissue and porphyrins produced by bacteria complicates the identification of autofluorescence sources within tissue and interpretation of data.^24^^,^^25^ Hyperspectral analysis techniques such as spectral unmixing allow separation of overlapping signals based on their individual fluorescence or absorption spectra.^26^ Although autofluorescence imaging is often performed on a microscopic level with cellular resolution, clinical wounds are typically millimeters to centimeters in diameter. Macroscopic fluorescence systems can capture autofluorescence and extract diagnostic information such as tissue malignancy^27^^,^^28^ or wound bioburden.^29^

HSI systems can be designed in different configurations using different optical components. Liquid crystal tunable filters are wavelength-dispersive devices with limited spectral resolution and switching time compared with interferometers or acousto-optical tunable filters (AOTF), but they offer high spatial resolution and do not involve moving parts.^19^ Although an endoscope-based hyperspectral reflectance and fluorescence system that uses an AOTF for spectral dispersion has been reported, the system requires two separate cameras for fluorescence and reflectance imaging.^28^ Some HSRI systems accomplish spectral dispersion on the illumination pathway using multiple LEDs^23^^,^^30^ or a supercontinuum laser.^31^ However, LED-based systems are limited by wavelength availability and require additional optical filtering to achieve narrower bandwidths,^23^ while supercontinuum lasers are typically fiber-based and not suitable for imaging over large FOVs without spatial scanning mechanisms, which increases the overall cost and acquisition time of the system.^32^

Here, a custom-built spectral scanning hyperspectral reflectance and autofluorescence system (HySAF) was designed with a large field of view and spatial resolution, as would be needed for preclinical evaluation of chronic and acute wounds. Wavelength dispersion was accomplished using a liquid crystal tunable filter (LCTF) capable of scanning across the visible light spectrum. The use of the liquid crystal tunable filter within the detection arm of HySAF facilitates both reflectance and fluorescence imaging with a single camera. Images were acquired at each wavelength setting of the LCTF using a custom Python script. The system was characterized by imaging USAF resolution targets at a range of wavelengths and calculating the field of view and spatial resolution. Spectral calibration was performed by obtaining dark current and 99% reflectance images and calculating the relative reflectance of sample images. Reference spectra for oxy- and deoxyhemoglobin were obtained from whole blood samples. Imaging of gold nanoparticles and immune cells evaluated the detection of sub-resolution particle effects, while autofluorescence performance was demonstrated by imaging a scorpion. Preliminary absorbance and fluorescence data from imaging of *in vivo* rat wounds illustrate HySAF’s tissue monitoring capabilities for further wound studies. This work presents a Python-controlled reflectance and autofluorescence hyperspectral imaging system optimized for wound monitoring and is sensitive to subresolution tissue changes in reflectance and fluorescence, demonstrating potential for both *in vitro* and *in vivo* investigations of wound-related research.

## 2 Materials and Methods

### 2.1 Optical Design of HySAF

**Fig. 1:**
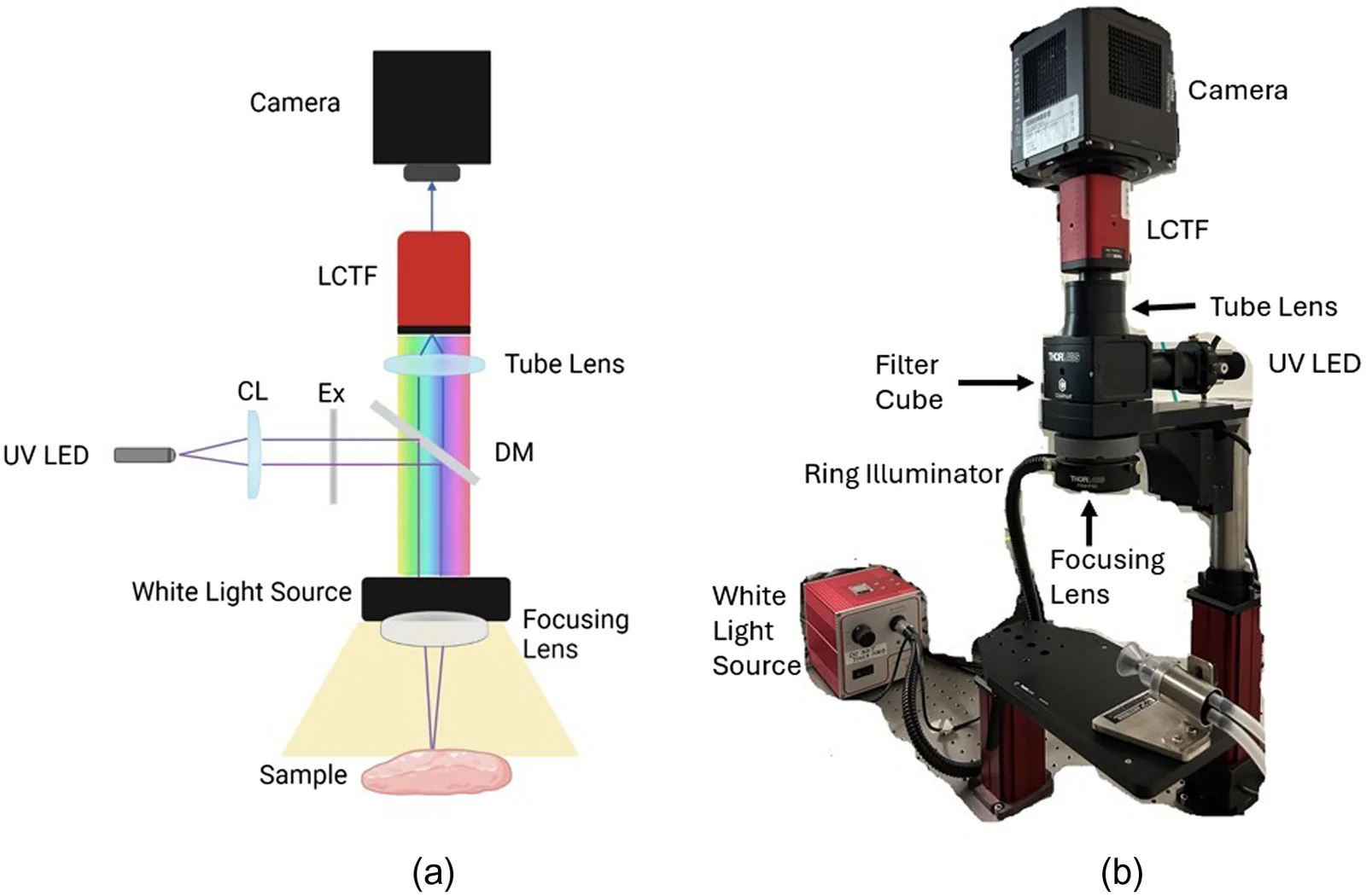
(a) Optical diagram of HySAF. LCTF = liquid crystal tunable filter, CL = collimation lens, Ex = fluorescence excitation filter, DM = dichroic mirror. (b) Photo of HySAF. The height of the system from the top of the camera to the breadboard table is approximately 2 ft (61 cm).

HySAF is a spectral scanning, macroscopic system (Fig. 1). The camera acquires a 2D spatial image of the sample as the tunable filter allows narrow bandwidths of light to reach the detector sequentially, resulting in a 3D dataset (x-y-λ). The microscope body is adapted from the Thorlabs Cerna Mini Microscope with epi-illuminator module (Thorlabs SFM, Newton, New Jersey, United States). The two illumination modes for hyperspectral reflectance and autofluorescence imaging can be easily switched. A broadband halogen lamp (Thorlabs OSL2) connected to a fiber ring illuminator (Thorlabs FRI61F50) that is suspended around the objective lens with a custom 3D-printed holder provides the reflectance illumination. The UV illumination arm consists of a 385 nm LED (Thorlabs M385L2) and collimation module connected to the Cerna filter cube holder, which contains a 357/44  nm excitation filter (Semrock FF01-357/44-25, Henrietta, New York, United States) and 409 nm dichroic beamsplitter (EdmundOptics 86-330, Barrington, New Jersey, United States). A 200 mm achromatic lens (Thorlabs AC254-200-A) is used to focus the UV light onto the sample and collect the fluorescence and reflected light from the autofluorescence and hyperspectral illumination, respectively. The Cerna microscope body allows the vertical position of the lens and ring illuminator to be adjusted and the stage itself can be adjusted, to account for the location of the sample surface. Both illumination modes share the same detection optics: a variable bandwidth liquid crystal tunable filter (Thorlabs Kurios-VB1) to switch through wavelengths from 420 to 730 nm, a 100 mm tube lens with 0.5× magnification (Thorlabs WFA4102), and a Kinetix22 sCMOS camera (Teledyne Photometrics) with a 2400×2400  pixel sensor and 13  μm effective pixel size. For fluorescence imaging, the LCTF is used as the emission filter.

### 2.2 Software Control of HySAF

A Python Jupyter Notebook was created to synchronously control the camera and LCTF with high temporal precision and control over settings. Using the software development kits (SDKs) for each device, we integrated the devices and specified the optimal settings and parameters for HSI. Simple functions to set and access attributes were used directly from the SDK, and custom functions were developed for complex operations such as optimizing camera exposure times and saving the raw data to a usable format.

**Fig. 2:**
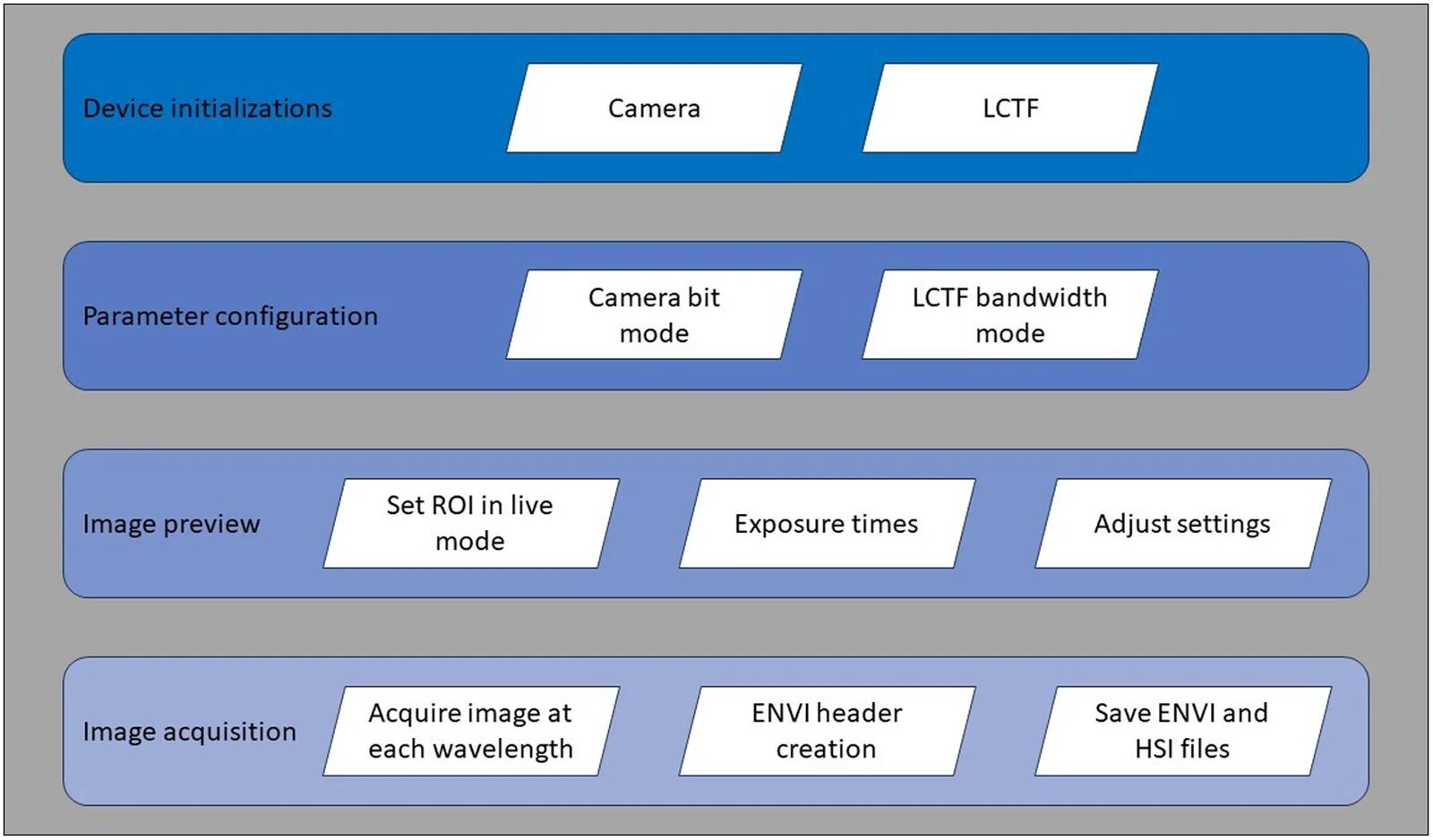
Structure diagram of Python Jupyter notebook control code for HySAF.

**Fig. 3:**
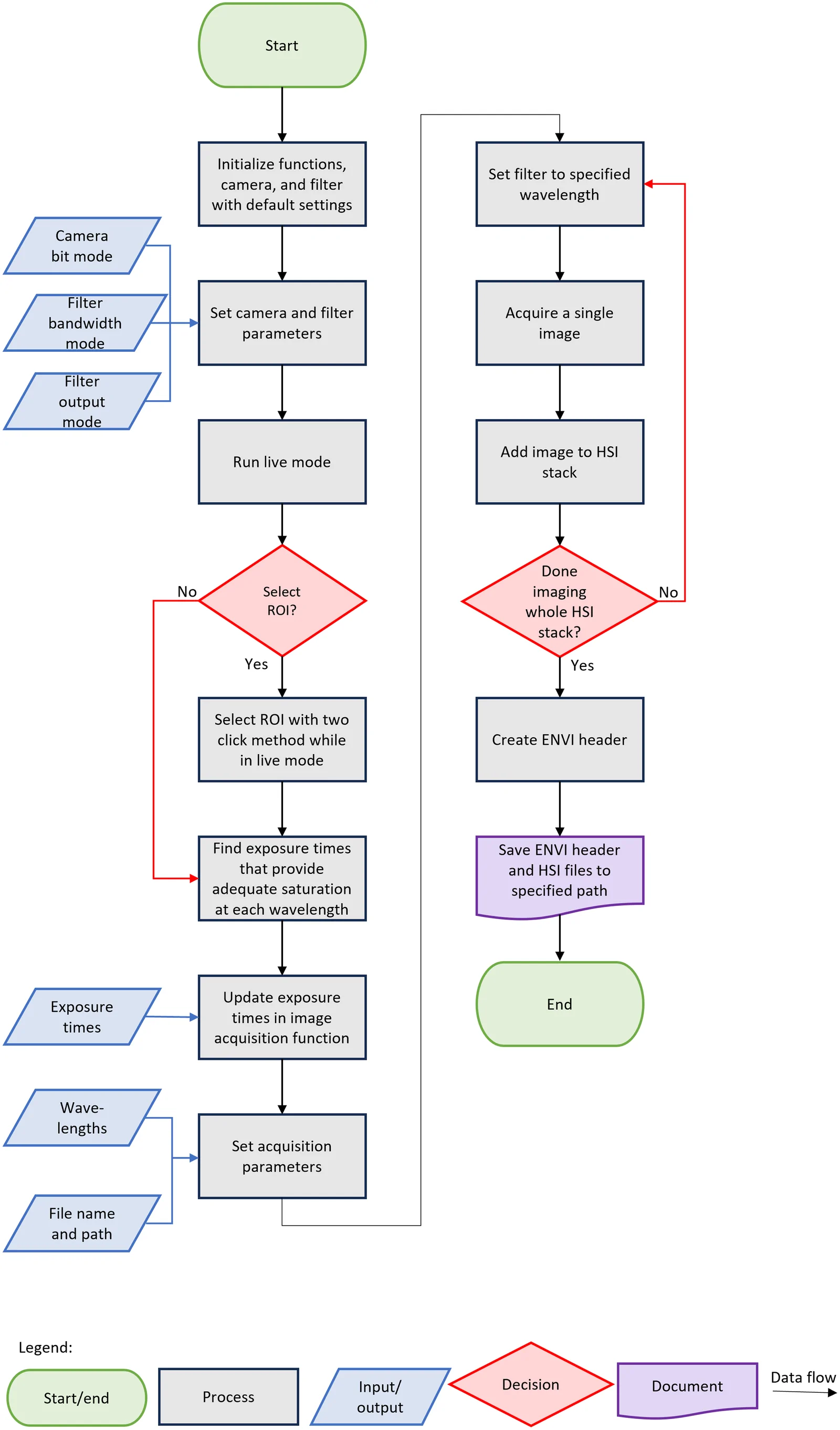
Flowchart showing the steps the HySAF acquisition code takes from system startup to image acquisition.

The Jupyter notebook was designed in an organized manner such that the overall acquisition process occurs in four steps, as inspired by the code structure outlined in Ref. 33. First, all devices and functions were initialized; next, additional settings or parameters were configured; third, the image was previewed; and finally, the image was acquired (Fig. 2). During the parameter configuration step, the camera bit mode and LCTF bandwidth mode were set with functions directly from the SDKs. The image was previewed with the custom live mode function, which allowed selection of a region of interest with a real-time histogram of pixel values. Variable exposure times were required to account for illumination variance at each wavelength. A custom function determines ideal exposure times for imaging using the 99% reflectance standard for white light reflectance images or a baseline fluorescence signal from an autofluorescent sample. The exposure times for all desired wavelengths were saved to a dictionary and used for all subsequent same-day image acquisitions. The range of desired wavelengths was passed as a list to a function that updates the filter and then acquires the corresponding image until the whole wavelength sequence is completed. The LCTF was kept in manual mode throughout the whole process to permit each distinct wavelength change. Once the acquisition procedure was completed, the image sequence was saved to the specified directory as a .raw file with an automatically created ENVI header to be opened and analyzed in a separate processing code (Fig. 3).

Due to its ease of use and ever-evolving analytical abilities, Python was selected as the primary programming language for this project. Using the software development kits (SDKs) provided by the manufacturers, the custom code allowed precise control over all device parameters and settings without the need to use additional hardware such as an external trigger, function generator, or third-party manager software that may not provide access to all the unique functions of a device. In addition, the HySAF Python program saves raw hyperspectral images and the metadata in an ENVI format for compatibility with a variety of analysis programs such as ImageJ, Matlab (MathWorks, Natick, Massachusetts, United States), or commercial hyperspectral analysis software such as IDCube (HSpeQ, St. Louis, Missouri, United States) or Evince (Middleton Spectral Vision, Middleton, Wisconsin, United States).

### 2.3 Characterization

Spatial resolution for the halogen source was measured with a cardstock 1× standard Air Force resolution target (AFT) from Thorlabs. The LCTF was set to the approximate middle wavelength (550 nm) and the resolution target was brought into focus before image capture. White light lateral resolution was determined by an edge-spread function (ESF) in Fiji (ImageJ).^34^ The FOV was determined by imaging a small metal ruler, and the ideal working distance was found by adjusting the height of the stage and z-position of the focusing lens until a clear, in-focus image was produced.

Lateral resolution for fluorescence mode imaging was measured by the same procedure as above using the UV LED as the illumination source, and a positive fluorescent AFT (EdmundOptics, 57-855) was used. The peak emission of the target with 385 nm excitation occurred at 540 nm, so that image was used for resolution analysis.

### 2.4 Reflectance Preprocessing

Because integration times at each wavelength were automatically optimized for each sample with the HySAF control software, the dark noise and white light calibration images were acquired with wavelength-matched exposure times. A calibrated reflectance standard (99% Spectralon) was imaged using consistent white illumination output to account for intensity nonuniformity across the field of view. The dark noise images were obtained with the light source turned off and the objective lens covered. All white light reflectance images were then run through a custom Python script to calibrate the reflectance of the sample image according to the equation

$$I_{\mathrm{refl}}=\frac{I_{\mathrm{raw}}-I_{\mathrm{dark}}}{I_{\mathrm{white}}-I_{\mathrm{dark}}},$$ (1)

where $I_{\mathrm{refl}}$ is the calibrated reflectance image, $I_{\mathrm{raw}}$ is the raw image of the sample, $I_{\mathrm{dark}}$ is the image of the dark current of the camera, and $I_{\mathrm{white}}$ is the image of the calibrated diffuse reflectance standard.^19^ Next, the absorbance image, $I_{\mathrm{abs}}$, was generated by taking the negative logarithm of the calibrated reflectance image, $I_{\mathrm{refl}}$, according to Eq. (2).^35^

$$I_{\mathrm{abs}}=-{\;\log\;}_{10}(I_{\mathrm{refl}}).$$ (2)

The mean reflectance and absorbance of the image pixels are calculated for each wavelength and plotted to show the absorbance and reflectance spectrum for each sample.

### 2.5 Validation Experiments

#### 2.5.1 Reflectance of whole blood

Visible hyperspectral image stacks of a drop of oxygenated and deoxygenated blood were acquired and compared with measured readings of dissolved oxygen (DO). Human whole blood (venous) was purchased (StemCell Technologies 70507.1). A dissolved oxygen meter (Fisher Scientific, 06-662-66) was used to measure the oxygen content of the blood prior to imaging. The meter probe was calibrated according to manufacturer instructions in the environment where imaging took place. A container of ∼15  mL of blood was exposed to pure $O_{2}$ gas until a stable maximum DO value was obtained. The DO probe had a sampling rate of 0.4 s but was allowed to stabilize for ∼30  s in the blood solution. Several drops were then placed on a Petri dish and covered with a glass slip to reduce glare for imaging and minimize contact with environmental air. The rest of the sample was deoxygenated with sodium dithionite (Sigma-Aldrich 101184-248) until the DO measurement reached a stable minimum. A drop was immediately transferred after the DO measurement to a slide, covered with a glass coverslip, and imaged within two minutes of the DO measurement. HySAF acquired all reflectance images from 440 to 730 nm at a bandwidth of 10 nm for a total of 30 images per stack. The exposure times are listed in Table S1 in the Supplementary Material.

#### 2.5.2 Detection of reflectance changes within cells due to gold nanoparticles

The HySAF system was used to sense gold nanoparticles in live-cell cultures. THP-1 cells were cultured in RPMI 1640 Medium (Gibco 11875135) with 10% fetal bovine (FBS, Gibco 05025001). THP-1 cells were seeded in T-25 flasks, which were vertically placed in an incubator set at 37^∘^C with 5% ${\mathrm{CO}}_{2}$ and 95% humidified air. Media was exchanged twice a week by replacing 2 mL of the suspended cell-media mixture with fresh culture medium. The cells were passaged once every 2 weeks, maintaining their viability above 85%. THP-1 monocytes were differentiated into M0 macrophages by adding 1  ng/mL of phorbol 12-myristate 13-acetate (ThermoFisher Scientific 356150010) per dish, promoting adherence to the dish surface. Bare gold nanostars (GNS) were synthesized following an established protocol.^36^ Hyperspectral images of (i) culture medium alone, (ii) M0 macrophages in culture medium, and (iii) M0 macrophages (in culture medium) incubated with GNS for 24 h, were acquired. All imaging was conducted directly on 2 mL of sample in 35 mm glass bottom dishes (MatTek). The exposure times used for the data collection are listed in Table S2 in the Supplementary Material.

To correct for system-specific artifacts and background interference, reference measurements were obtained from an empty dish, a 99% reflectance standard, and a dark optical blocker simulating 0% reflectance.

$$I_{\mathrm{refl}}=(\frac{I_{\mathrm{raw}}-I_{\mathrm{dark}}}{I_{\mathrm{white}}-I_{\mathrm{dark}}}-\frac{I_{\mathrm{empty}}-I_{\mathrm{dark}}}{I_{\mathrm{white}}-I_{\mathrm{dark}}})\times 0.99.$$ (3)

The reflectance of the dataset was calculated using the formula given by Eq. (3) where $I_{\mathrm{refl}}$ are the corrected reflectance values of the sample (ranging from 0 to 1) for each spatial pixel, ([1 ×1 ×30]), $I_{\mathrm{raw}}$ are the raw reflectance intensity values for the sample directly imaged by the system, $I_{\mathrm{dark}}$ are the reflectance intensity values of a dark background under no illumination, $I_{\mathrm{empty}}$ are the reflectance intensity values for the empty dish with no sample. Similarly, the absorbance values were calculated as given by Eq. (2).

Validation of spectral properties of the solutions was performed using UV-Vis spectrophotometry. Three samples, culture medium alone, a mixture of cells and culture medium, and a mixture of cells, culture medium, and GNS, were scraped from the imaging dishes after hyperspectral imaging. The collected samples were transferred into 15 mL disposable test tubes for UV-Vis spectral measurements. Subsequently, 200  μL of each sample was pipetted into a clear 96-well plate (Thermo Fisher Scientific, Catalog No. 15045) and scanned using a Tecan Infinite 200 Pro microplate reader over a wavelength range of 450 to 900 nm with a spectral resolution of 1 nm.

#### 2.5.3 Autofluorescence of scorpion

Autofluorescence images of a locally sourced deceased striped bark scorpion were captured to demonstrate the fluorescence imaging capabilities of HySAF and the sensitivity of the camera. The 385 nm LED was used to illuminate a large portion of the scorpion’s body at an illumination power of ∼12  mW. Fluorescent images were acquired as the LCTF swept through 22 wavelengths from 440 to 650 nm at 18 nm bandwidth. Camera exposure times for the following wavelength ranges were used: 900 ms for 440 to 460 nm, 400 ms for 470 to 480 nm, and 490 to 650 at 300 ms.

#### 2.5.4 Reflectance and autofluorescence of rat wounds

A pilot study to image rat wounds*in vivo* with HySAF demonstrated the suitability of the system for small animal tissue monitoring. The study was performed in accordance with TAMU IACUC protocol 2024-0084. Two eight-week-old healthy Lewis rats (one male and one female) were anesthetized with isofluorane, and the shaved skin on the back was imaged using HySAF’s reflectance and fluorescence modes to obtain a baseline. Each rat then underwent two full-thickness punch biopsies to create four 6 mm wounds behind the shoulder blades. The wounds were allowed to heal naturally for 21 days, after which the rats were humanely sacrificed. White light reflectance and autofluorescence imaging occurred immediately after wound creation and again on Days 7 and 21. Any hair that had grown back and obscured the wounds and surrounding skin was shaved prior to imaging. During imaging, the rats were placed on a warming pad on the stage while the inhaled anesthetic was delivered through a nose cone. The rats were positioned the same way for each imaging day to ensure consistency: they were placed on their belly with the limbs gently stretched out to the sides so that their backs created a flat surface to minimize surface distortion in the images. Respiratory rate and depth of anesthesia (toe pinch) were assessed every 15 min while the rat was anesthetized. The height of the stage was adjusted until the skin surface was located at the approximate working distance of the objective lens (190 mm) and the wound appeared in focus on the live image preview. All ambient light sources were turned off, and the illumination sources were set to a consistent output level. The halogen white light source was set to 10% of its maximum intensity ($<1\;\mathrm{mW}/{\mathrm{cm}}^{2}$ optical power at sample), and the 385 nm LED had a sample power output of $∼10\;\mathrm{mW}/{\mathrm{cm}}^{2}$. On each imaging day, camera exposure times for white light (440 to 730 nm) and 385 nm illumination (420 to 700 nm) were determined using the automatic exposure time optimizer mode within HySAF’s Python acquisition code. Hyperspectral image acquisition time for white light hypercubes was about 3 to 5 s and autofluorescence hypercubes took around 15 to 20 s due to higher camera exposure times. The average exposure times used for each wavelength across imaging days are shown in Tables S3 and S4 in the Supplementary Material. In a Python script, white light images were converted to reflectance images using their corresponding 99% calibration and dark noise images according to Eq. (1) and absorbance images were calculated according to Eq. (2). For both absorbance and fluorescence hyperspectral images, the mean intensity was calculated for a region of interest (ROI) containing the center of the wound bed, or a section of healthy skin for the baseline measurements. As the rats were anesthetized, only minimal motion artifacts were observed due to respiration, and these artifacts did not affect the calculation of the absorbance and fluorescence means or standard deviations because the selected ROI excluded motion-corrupted data. Then, the mean fluorescence and absorbance values from the four wounds and two baselines were averaged together by timepoint and plotted with RStudio, including the standard deviation between the wounds and baseline skin.

## 3 Results

### 3.1 HySAF Optical Performance

**Fig. 4:**
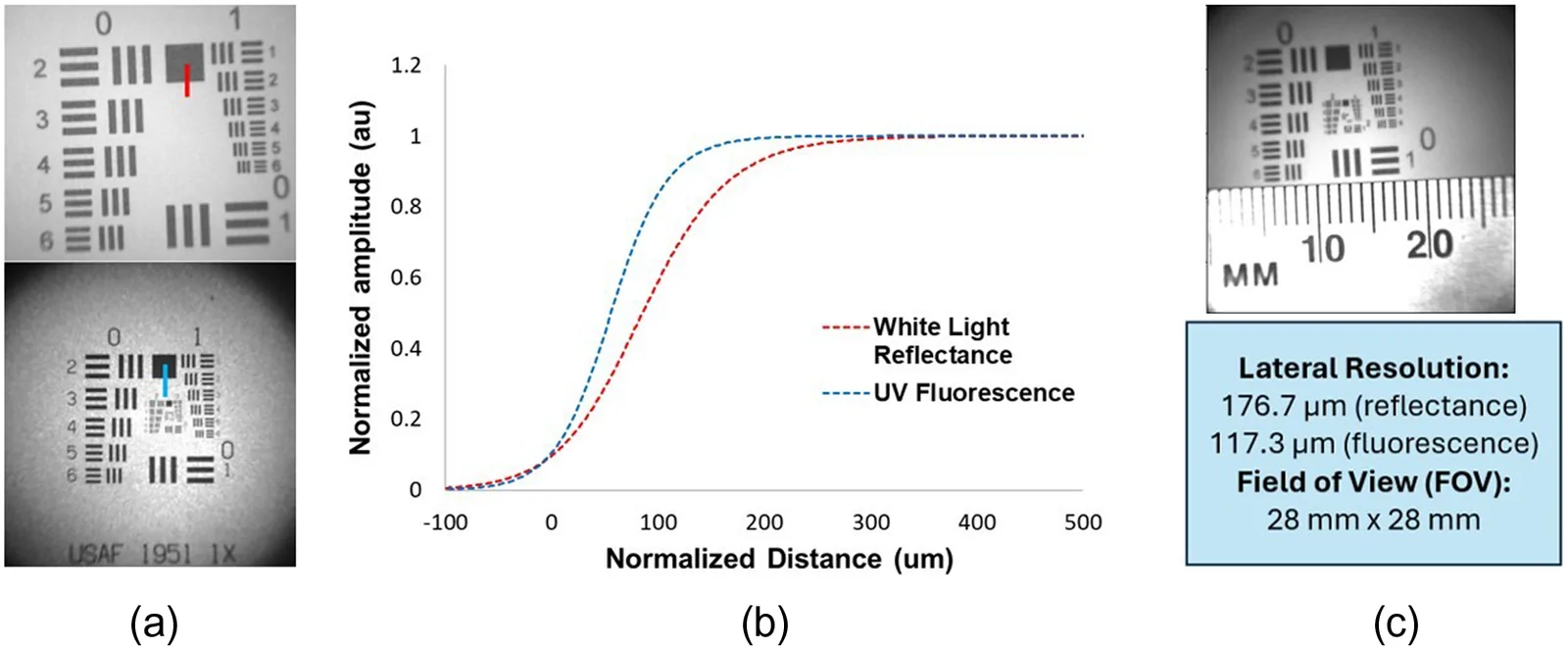
(a) Air Force resolution target images with white light reflectance (top) and UV fluorescence (bottom). (b) Comparison of measured edge-spread function (ESF) of the resolution targets. The location of the ESF is denoted on the images with a red or blue line. (c) HySAF field of view (FOV) with ruler and resolution target.

First, the optical performance of HySAF was characterized. The overall magnification of the system is 0.5×. The lateral resolution for white light illumination and reflectance imaging was determined to be 176.7  μm (Fig. 4) for a FOV of 28 ×28  mm, and a working distance of about 190 mm.

UV-illumination resolution was determined to be 117.3  μm and the FOV is the same, 28 ×28  mm (Fig. 4). A customized acquisition program in Python was developed for full control and compatibility of the hardware that was sourced from multiple manufacturers. This was advantageous over integrating the illumination control, LCTF, and camera into an existing hardware-managing software such as MicroManager because the customized code allowed full control and automation of the parameters for each hardware component and synchronization among them.

### 3.2 HySAF Imaging of Oxygenated and Deoxygenated Blood

**Fig. 5:**
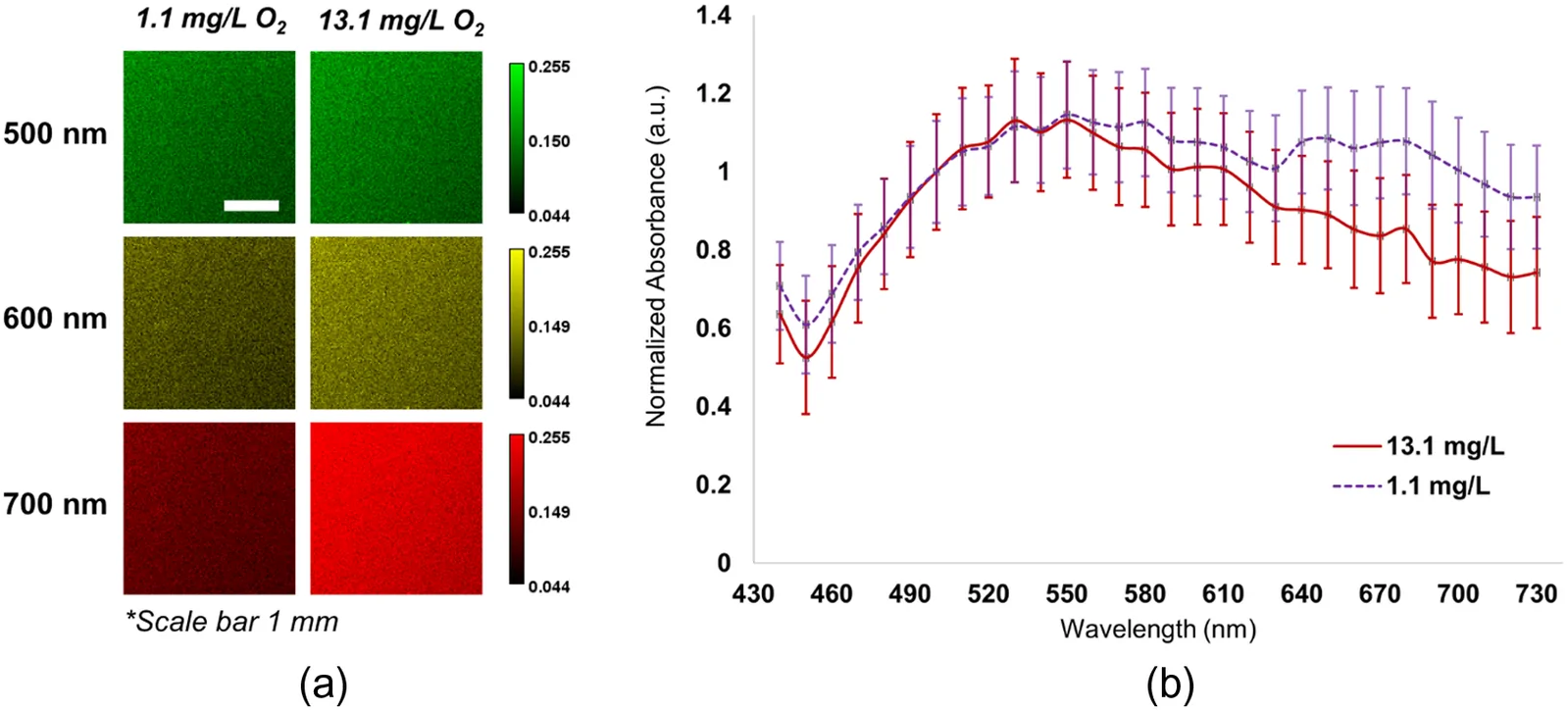
(a) Representative raw reflectance images of deoxygenated and oxygenated blood samples. Images are false colored and set to uniform brightness and contrast scales. Scale bar is 1 mm. (b) Calculated average absorbance and standard deviation values for the oxygenated and deoxygenated blood samples normalized to 500 nm.

Representative false-colored reflectance images acquired with HySAF show similar reflectance intensities of deoxygenated and oxygenated blood at 500 nm and differences at 600 and 700 nm (Fig. 5). The DO meter measured 13.1  mg/L of oxygen in the sample considered oxygenated and 1.1  mg/L in the deoxygenated sample. The absorbance of the two blood samples was calculated from the HySAF-measured reflectance images, and the absorption spectra were normalized to 500 nm [Fig. 5(b)]. The standard deviation represents the variation between pixel intensities within the selected ROI of the blood samples. The absorption of the oxygenated and deoxygenated blood samples was similar from 430 to 550 nm [Fig. 5(b)]. At wavelengths greater than 550 nm, the deoxygenated blood had higher absorbance than the oxygenated blood [Fig. 5(b)].

### 3.3 HySAF Detects Reflectance Changes due to Nanoparticles

**Fig. 6:**
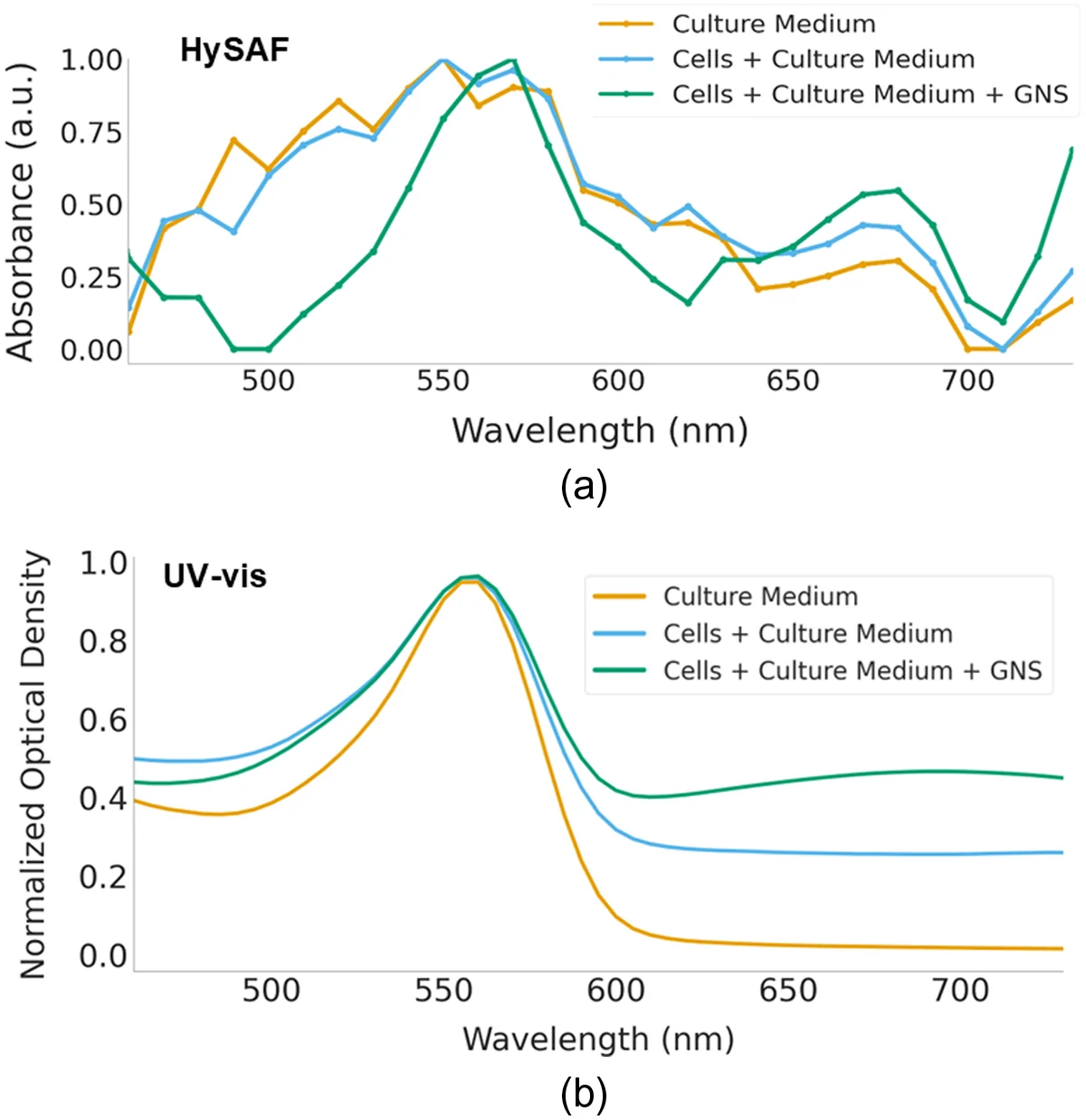
(a) HySAF calculated absorbance spectra for cell culture medium alone [orange], THP–1 derived M0 macrophages in culture medium (uninteracted) [blue] and after 24 h of interaction with bare GNS [green]. (b) UV–Vis spectroscopy measurements for spectra for cell culture medium alone [orange], THP–1 derived M0 macrophages in culture medium (uninteracted) [blue] and after 24 h of interaction with bare GNS [green].

The reflectance and absorbance spectra were compared for culture medium, cells (M0 macrophages) in culture medium, and cells incubated with gold nanostars (GNS) to evaluate the capabilities of the system to detect absorbance changes due to particles smaller than the resolution of the system. Reflectance measurements showed that cells exhibited higher reflectance than culture medium alone (∼5% versus ∼1% baseline across wavelengths), while the addition of GNS further increased reflectance to ∼20% to 30% across the visible range (Fig. S1 in the Supplementary Material). To evaluate changes in absorption spectra, HySAF reflectance images were acquired and used to compute normalized absorbance spectra for culture medium, cells in medium, and cells incubated with GNS (Fig. 6). All three groups displayed a prominent absorption peak centered around 550 to 560 nm. Compared with the culture medium control, the addition of cells introduced a vertical upward shift in the baseline absorbance [Fig. 6(a)], a trend highly consistent with UV–Vis plate-reader profiles [Fig. 6(b)]. In comparison to the cells in the media group, the introduction of GNS altered the primary peak dynamics and resulted in a distinctly elevated absorbance at longer wavelengths between 650 and 730 nm [Fig. 6(a)], capturing the characteristic red-shifted localized surface plasmon resonance (LSPR) signature of the gold nanostars. UV–Vis plate–-reader absorption spectra of the same samples show similar absorption spectra of culture medium and cells and increased absorption between 650 and 700 nm for cells with GNS [Fig. 6(b)]. For both HySAF and UV–Vis plate-reader data, spectral curves were normalized using a localized min–max transformation where the minimum and maximum absorbance bounds were evaluated exclusively between 450 and 730 nm. This scaling factor was applied uniformly to both the mean absorbance and standard deviation arrays across all evaluated wavelengths. Resultant negative values outside the primary normalization window were truncated to zero to ensure physical boundary constraints.

### 3.4 HySAF Performance for Imaging Autofluorescence of Scorpion

**Fig. 7:**
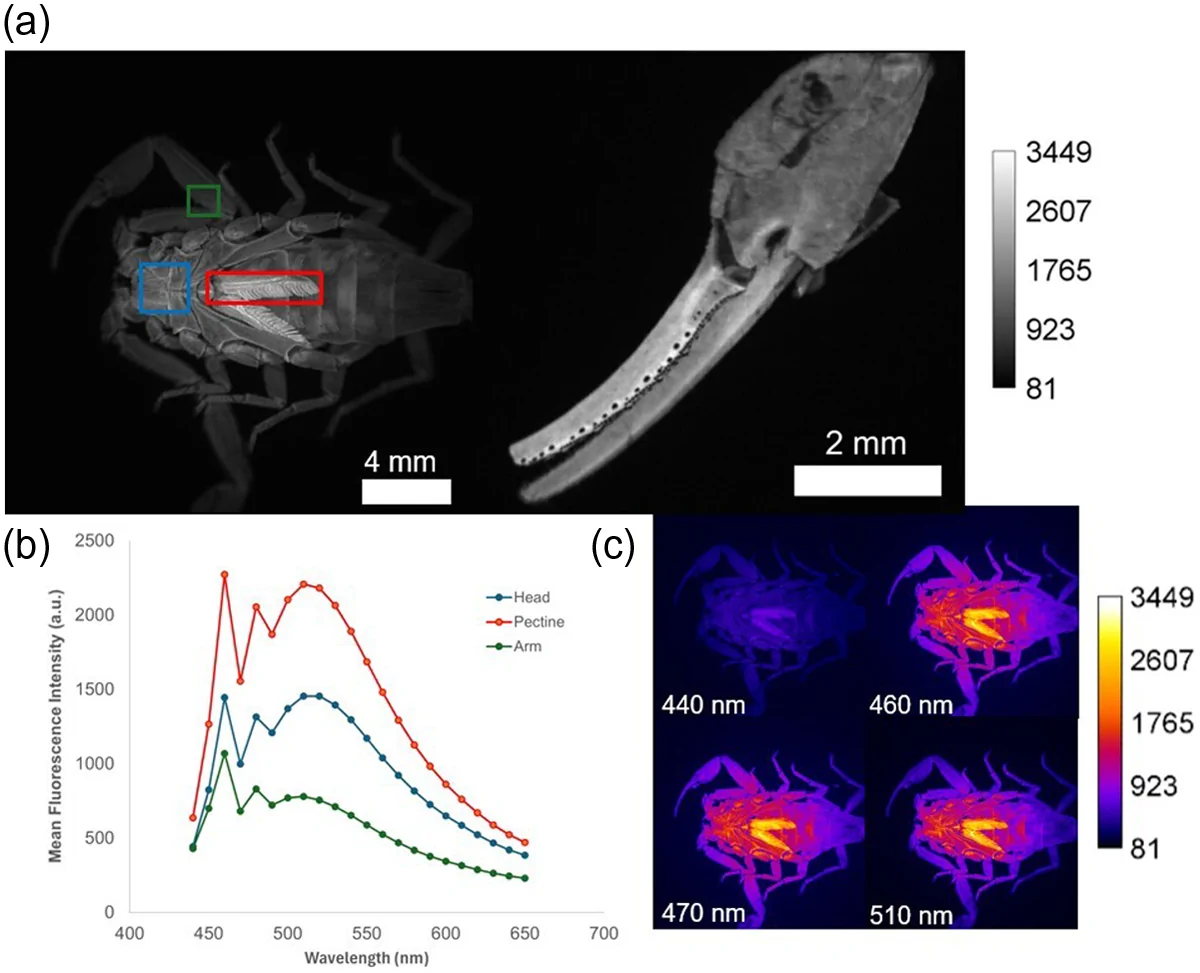
(a) Fluorescence image of scorpion (left, ventral view) and close-up of pincer showing details of fingers (right) at 540 nm. Colored boxes correspond to regions used to calculate mean fluorescence intensity spectra (b). (c) Fluorescent intensity images at 440, 460, 470, and 510 nm of the scorpion body false colored to a heat map.

Autofluorescence imaging of the scorpion with 385 nm illumination demonstrated HySAF’s ability to capture fluorescence images and resolve small features of interest at a macroscopic level (Fig. 7). Representative fluorescence images at 540 nm of the scorpion body and pincer demonstrate the fluorescence mode resolution and dynamic range to resolve tissue autofluorescence [Fig. 7(a)]. A region of interest focused on the scorpion’s pincer illustrates how the fixed finger exhibits higher autofluorescence intensity and HySAF’s ability to resolve small details such as the ridges along the edge of the finger used to grasp prey. The HySAF-measured fluorescence images and quantified spectra of selected regions, the head (blue), arm (green), and a pectine (feather-like sensory organ, red) show the mean fluorescence intensity of these different sections of the scorpion’s body vary in magnitude across wavelength, with the pectine having the highest intensity, followed by the head and arm with the lowest intensity [Figs. 7(b) and 7(c)].

### 3.5 HySAF Imaging of In Vivo Rat Wounds

**Fig. 8:**
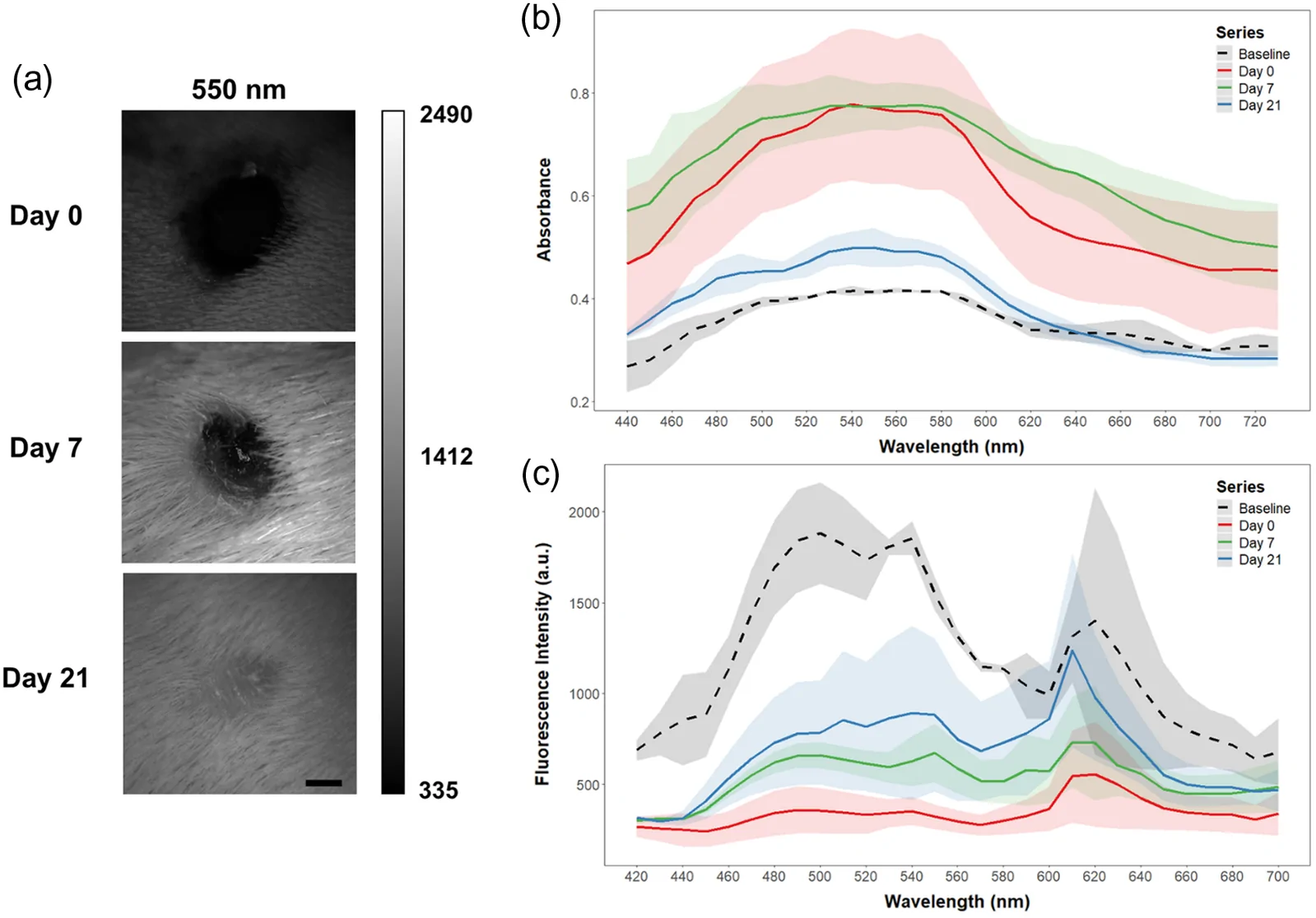
(a) Representative raw white light images of rat wounds at 550 nm acquired on Days 0, 7, and 21. Scale bar represents 2 mm. (b) Mean absorbance spectra with standard deviation (shaded area) for wound (n=4 wounds) and baseline, prewounded tissue (n=2 subjects). (c) Mean fluorescence intensity spectra with standard deviation for wound (n=4 wounds) and baseline (n=2 subjects).

Preliminary *in vivo* data of white light reflectance and 385 nm autofluorescence images of a rat wound capture biological changes in tissue optical properties over time (Fig. 8). The wound bed absorbance spectra exhibited overall higher absorbance at Days 0 and 7 with an exaggerated peak in the 500 to 600 nm range, which is more pronounced at Day 0 [Fig. 8(b)]. The standard deviation of the absorption spectra of the rat wounds is larger on Day 0 on than the other days, suggesting heterogeneity across rats and wounds, which may be due to different amounts of effluent. By Day 21, the spectral shape and amplitude closely resemble the prewounding baseline spectra. This trend of re–normalization of spectral shape over time is also seen in the autofluorescence spectra [Fig. 8(c)]. Here, the baseline spectra show several distinct peaks in fluorescence intensity of normal rat skin, notably at 480 to 500 nm, 540 nm, and 620 nm. Following wound creation on Day 0, these signals are largely lost, but gradually return over the time course due to reepithelialization of the wound bed. However, the magnitude of intensity of the wounded regions from 420 to 580 nm at Day 21 is less than the baseline, whereas the fluorescence intensity of the wounded tissue in the 590 to 700 nm range by day 21 is similar in magnitude to the baseline measurements.

## 4 Discussion and Conclusion

Hyperspectral imaging systems are noninvasive optical imaging devices with many potential biomedical applications at the macro- and microscopic levels, including wound assessment and monitoring. Spectral imaging resolves biological information, which cannot be observed unaided, such as tissue perfusion and oxygenation. Our goal was to build a customizable multimodal spectral imaging system to capture diffuse reflectance and fluorescence images of samples of varying sizes and optical properties. In addition to characterizing the optical performance of HySAF, validation experiments were performed to evaluate the capabilities of HySAF to detect absorption differences of oxygenated and deoxygenated blood, reflectance contrast due to particles smaller than the spatial resolution by imaging cells with and without nanoparticles, macroscopic tissue autofluorescence imaging using a scorpion, and absorbance and autofluorescence measurements of a rat wound model. Paired with a sensitive sCMOS camera, HySAF is able to detect weak signals such as nanoparticle absorbance in cells (Fig. 6) and autofluorescence due to tissue fluorophores (Fig. 8) at a low magnification. The system was built using a customizable microscope body (Thorlabs Cerna) to facilitate modifications to the optical path, such as implementing and testing new hardware or changing magnification.

HySAF’s optical performance with a large FOV and ample vertical space (19 mm) to maneuver is well suited for both *in vitro* and small animal *in vivo* imaging studies. In addition, the objective lens’ 200 mm focal length improves the depth of field (DOF), which is important when imaging nonflat surfaces such as rat skin. The improvement of resolution from 176 to 117.3  μm with UV illumination is reasonable, as the halogen source is significantly red-shifted and spatial resolution will deteriorate at longer wavelengths and across a wide-spectrum due to focal length shift of the achromatic focusing lens.^37^ Although HySAF’s magnification (0.5×) and spatial resolution are low compared to other benchtop hyperspectral systems^25^^,^^38^^–^^40^, HySAF’s field of view is optimized for wound and tissue imaging; therefore, cellular resolution is not required for target applications. There are some limitations of HySAF and its software control developed using Python. Although our approach offers better spatial resolution than systems with line or point-scanning configurations, the LCTF has slower switching speeds and a spectral range compared with AOTFs or Fabry-Perot interferometers, which hinders acquisition time and restricts any biologically relevant data obtained to a smaller wavelength range.^19^^,^^23^ The system incorporates expensive components (sCMOS camera, LCTF, and customizable microscope body) and must be stabilized to prevent motion artifacts, so current implementation into the clinic is not possible. The Python Jupyter notebook lacks an intuitive user interface that is standard in most commercial hyperspectral software.

To ensure that HySAF is capable of resolving biologically relevant spectral differences in tissue optical properties, HySAF was used to image the diffuse reflectance of oxygenated and deoxygenated whole blood as the oxygenation state changes its reflection and absorption profiles. Then, HySAF was used to acquire both reflectance and autofluorescence images of healing rat wounds. First, absorbance spectra were acquired of blood samples with dissolved oxygen (DO) measurements of 1.1 and 13.1  mg/L. Although the amount of oxygen dissolved in blood plays a smaller role in oxygen transport in comparison to hemoglobin binding, it is still an appropriate gauge for the presence of oxygen in a blood sample.^41^ The HySAF-measured absorption spectra relatively match published spectra of the absorption coefficient of oxy- and deoxyhemoglobin, where double peaks around 550 nm are visible for oxygenated blood, and deoxygenated blood has a higher absorbance than oxygenated blood from 600 to 730 nm [Fig. 5(b)].^42^^–^^44^ Imaging of *in vivo* wounds demonstrated differing spectral shapes and magnitudes for both absorbance and autofluorescence data relative to baseline over the time course (Fig. 8). In addition, the spectra exhibited local peaks that will be further investigated to identify corroboration with tissue biomarkers.^45^^–^^47^ These findings establish HySAF’s capability of monitoring optical changes in wounds.

HySAF captured both cellular-induced alterations in media absorption and pronounced reflectance enhancement due to nanoparticle presence within cells [Figs. 6(a) and 6(c), Fig. S1 in the Supplementary Material]. The HySAF-detected absorption spectra matched the spectral trends obtained from a standard UV-Vis spectrometer [Figs. 6(b) and 6(d)]. The agreement between HySAF and UV-Vis spectroscopy confirmed the HySAF-measured absorbance measurements. This highlights the capability of HySAF to detect biological variations and robust nanoparticle-induced signatures, even when the underlying materials that generate contrast are smaller than the spatial resolution.^14^^,^^17^ This makes the platform suitable for rapid, wide-field screening before more localized, high-resolution imaging, offering a practical bridge between benchtop spectroscopy and microscopic analysis.

In addition, HySAF can identify locations with different concentrations of a fluorophore as seen in Fig. 7 with certain areas of the scorpion’s body exhibiting higher intensity than others. The intensity of the autofluorescent signal was brightest from 460 to 560 nm, which aligns with the bright cyan-green glow visible to the human eye when the scorpion is exposed to UV light.^48^ Illumination with the UV LED as opposed to the white source resulted in superior resolution of small details on the scorpion’s body with the measured edge-spread resolution of 117.3  μm (Fig. 4). Keller et al. were able to differentiate between healthy and cancer cell types using endogenous autofluorescence imaging of resected breast tissue^27^ and Kesavan et al. classified wound bacteria as Gram-positive or negative based on macroscopic autofluorescent signal,^29^ which illustrates the potential of macroscopic imaging technologies to capture signals originating from sub-cellular particles and correlate them with optical property changes in biological samples.

A macroscopic reflectance and autofluorescence hyperspectral imaging system was designed and built with customizable hardware and Python-based acquisition software for imaging biological samples and tissue absorption and fluorescence. The performance results of HySAF indicate that it could be used for monitoring wounds *in vivo* and identifying notable biomarkers such as endogenous fluorophores or tissue oxygenation. Future directions include further system validation with turbid fluorescence and scattering phantom imaging as well as continuing *in vivo* rat wound studies to investigate biological correlation with spectral data.

## Supplementary Material

10.1117/1.JBO.31.9.096003.s01

## Data Availability

Acquisition and preprocessing code is available on GitHub. Data available upon reasonable request to the corresponding author.

## References

[1] Sen C. K., “Human wound and its burden: updated 2022 compendium of estimates,” (2023). 10.1089/wound.2023.0150 PMC10615092 37756368

[r2] Falanga V. et al., “Chronic wounds,” Nat. Rev. Dis. Prim. 8(1, 8), 1–21 (2022). 10.1038/s41572-022-00377-3 PMC10352385 35864102

[r3] Armstrong D. G. Boulton A. J. Bus S. A., “Diabetic foot ulcers and their recurrence,” New Engl. J. Med. 376, 2367–2375 (2017). 10.1056/NEJMra1615439 28614678

[r4] Harding K. G. Morris H. L. Patel G. K., “Science, medicine, and the future: healing chronic wounds,” BMJ : Br. Med. J. 324, 160–163 (2002). 10.1136/bmj.324.7330.160 11799036 PMC1122073

[5] Smet S. et al., “The measurement properties of assessment tools for chronic wounds: a systematic review,” Int. J. Nurs. Stud. 121 (2021). 10.1016/j.ijnurstu.2021.103998 34237439

[6] Nussbaum S. R. et al., “An economic evaluation of the impact, cost, and medicare policy implications of chronic nonhealing wounds,” Value Health 21, 27–32 (2018). 10.1016/j.jval.2017.07.007 29304937

[7] Li W. W. et al., “Vascular assessment of wound healing: a clinical review,” Int. Wound J. 14, 460–469 (2017). 10.1111/iwj.12622 27374428 PMC7950183

[8] Bowler P. G. Duerden B. I. Armstrong D. G., “Wound microbiology and associated approaches to wound management,” Clin. Microbiol. Rev. 14(2), 244–269 (2001). 10.1128/CMR.14.2.244-269 11292638 PMC88973

[9] Thiem D. G. et al., “New approach to the old challenge of free flap monitoring—Hyperspectral imaging outperforms clinical assessment by earlier detection of perfusion failure,” J. Pers. Med. 11, 1101 (2021). 10.3390/jpm11111101 34834453 PMC8625540

[10] Ramirez-GarciaLuna J. L. et al., “Is my wound infected? A study on the use of hyperspectral imaging to assess wound infection,” Front. Med. 10, 1165281 (2023). 10.3389/fmed.2023.1165281 PMC10483069 37692790

[11] Li J. Chen J. Kirsner R., “Pathophysiology of acute wound healing,” Clin. Dermatol. 25, 9–18 (2007). 10.1016/j.clindermatol.2006.09.007 17276196

[12] Petkovic M. et al., “Immunomodulatory properties of host defence peptides in skin wound healing,” Biomolecules 11, 952 (2021). 10.3390/biom11070952 34203393 PMC8301823

[13] Sullivan S. R. Engrav L. H. Klein M. B., “Approach to acute wound management,” in ACS Surgery: Principles and Practice, Fink M. P. et al., Eds., ch. 7, 6th ed., pp. 102–126, WebMD Professional Publishing, New York (2007).

[14] Calin M. A. et al., “Hyperspectral imaging in the medical field: present and future,“ Appl. Spectrosc. Rev. 49, 435–447 (2013). 10.1080/05704928.2013.838678

[15] Saiko G. et al., “Hyperspectral imaging in wound care: a systematic review,” Int. Wound J. 17, 1840–1856 (2020). 10.1111/iwj.13474 32830443 PMC7949456

[16] Zuzak K. J. et al., “Visible reflectance hyperspectral imaging: characterization of a noninvasive, in vivo system for determining tissue perfusion,” Anal. Chem. 74, 2021–2028 (2002). ANCHAM 0003-2700 10.1021/ac011275f 12033302

[17] Mangotra H. et al., “Hyperspectral imaging for early diagnosis of diseases: a review,” Expert Syst. 40, e13311 (2023). 10.1111/exsy.13311

[r18] Calin M. A. Manea D. Parasca S. V., “Comparison of spectral angle mapper and support vector machine classification methods for mapping skin burn using hyperspectral imaging,” Proc. SPIE 10677, 106773P (2018). 10.1117/12.2319267

[19] Lu G. Fei B., “Medical hyperspectral imaging: a review,” J. Biomed. Opt. 19, 010901 (2014). JBOPFO 1083-3668 10.1117/1.JBO.19.1.010901 24441941 PMC3895860

[20] Denstedt M. et al., “Hyperspectral imaging as a diagnostic tool for chronic skin ulcers,” Proc. SPIE 8565, 85650N (2013). 10.1117/12.2001087

[21] Daeschlein G. et al., “Hyperspectral imaging as a novel diagnostic tool in microcirculation of wounds,” Clin. Hemorheol. Microcirc. 67(3-4), 467–474 (2017). 10.3233/CH-179228 28885215

[22] Jayachandran M. et al., “Critical review of noninvasive optical technologies for wound imaging,” Adv. Wound Care 5, 349–359 (2016). 10.1089/wound.2015.0678 PMC4991615 27602254

[23] Tran M. H. Fei B., “Compact and ultracompact spectral imagers: technology and applications in biomedical imaging,“ J. Biomed. Opt. 28, 040901 (2023). 10.1117/1.JBO.28.4.040901 37035031 PMC10075274

[24] Zhang S. et al., “Dual-modality hyperspectral microscopy for transmission and fluorescence imaging,” Opt. Contin. 1(11), 2404 (2022). 10.1364/OPTCON.469040

[25] Deal J. et al., “Identifying molecular contributors to autofluorescence of neoplastic and normal colon sections using excitation-scanning hyperspectral imaging,” J. Biomed. Opt. 24, 021207 (2018). JBOPFO 1083-3668 10.1117/1.JBO.24.2.021207 30592190 PMC6307688

[26] Schultz R. A. et al., “Hyperspectral imaging: a novel approach for microscopic analysis,” Cytometry 43(4), 239–247 (2001). 10.1002/1097-0320 11260591

[27] Keller M. D. et al., “Autofluorescence and diffuse reflectance spectroscopy and spectral imaging for breast surgical margin analysis,” Lasers Surg. Med. 42, 15–23 (2010). LSMEDI 0196-8092 10.1002/lsm.20865 20077490

[28] Martin M. E. et al., “An AOTF-based dual-modality hyperspectral imaging system (DMHSI) capable of simultaneous fluorescence and reflectance imaging,” Med. Eng. Phys. 28, 149–155 (2006). 10.1016/j.medengphy.2005.04.022 15955718

[29] Kesavan R. Sasikumar C. S., “Clinical significance of a novel imaging device to evaluate infection on wounds: Performance comparison with culture method and metagenome sequencing,” J. Wound Manage. 23, 182–192 (2022). 10.35279/jowm2022.23.03.06

[30] Islam K. Ploschner M. Goldys E. M., “Multi-LED light source for hyperspectral imaging,” Opt. Express 25(26), 32659–32668 (2017). OPEXFF 1094-4087 10.1364/OE.25.032659

[31] Torabzadeh M. et al., “Hyperspectral imaging in the spatial frequency domain with a supercontinuum source,” J. Biomed. Opt. 24, 071614 (2019). JBOPFO 1083-3668 10.1117/1.JBO.24.7.071614 31271005 PMC6995957

[32] Chen W. Chen Z. Xing D., “Optical coherence hyperspectral microscopy with a single supercontinuum light source,” J. Biophotonics 14, e202000491 (2021). JBOIBX 1864-063X 10.1002/jbio.202000491 34004076

[33] Wang W. et al., “Development of software for spectral imaging data acquisition using LabVIEW,” Comput. Electron. Agric. 84, 68–75 (2012). CEAGE6 0168-1699 10.1016/j.compag.2012.02.010

[34] Schindelin J. et al., “Fiji: an open-source platform for biological-image analysis,” Nat. Methods 9(7), 676–682 (2012). NMAEA3 1548-7091 10.1038/nmeth.2019 22743772 PMC3855844

[35] Clancy N. T. et al., “Surgical spectral imaging,” Med. Image Anal. 63, 101699 (2020). 10.1016/j.media.2020.101699 32375102 PMC7903143

[36] Vu N. N. et al., “High precision automated synthesis of surface-enhanced (Resonance) raman nanotags,” ACS Sens. 10, 3515–3529 (2025). 10.1021/acssensors.5c00068 40299759

[37] Leung C. Donnelly T. D. Phys A. J., “Measuring the spatial resolution of an optical system in an undergraduate optics laboratory,” Amer. J. Phys. 85, 429–438 (2017). AJPIAS 0002-9505 10.1119/1.4979539

[38] Halicek M. et al., “Tumor detection of the thyroid and salivary glands using hyperspectral imaging and deep learning,” Biomed. Opt. Express 11(3), 1383–1400 (2020). BOEICL 2156-7085 10.1364/BOE.381257 32206417 PMC7075628

[r39] Hedde P. N. et al., “Phasor-based hyperspectral snapshot microscopy allows fast imaging of live, three-dimensional tissues for biomedical applications,” Commun. Biol. 4(1), 1–11 (2021). 10.1038/s42003-021-02266-z 34117344 PMC8195998

[40] Bertani F. R. et al., “Living matter observations with a novel hyperspectral supercontinuum confocal microscope for VIS to near-IR reflectance spectroscopy,” Sensors 13, 14523–14542 (2013). 10.3390/s131114523 24233077 PMC3871062

[41] Powell F. L. Wagner P. D. West J. B., “Ventilation, blood flow, and gas exchange,” in Murray and Nadel’s Textbook of Respiratory Medicine: Volume 1,2, Courtney Broaddus V. et al., Ed., 6th ed., pp. 44–75 (2016).

[42] Bjorgan A. Milanic M. Randeberg L. L., “Estimation of skin optical parameters for real-time hyperspectral imaging applications,” J. Biomed. Opt. 19, 066003 (2014). JBOPFO 1083-3668 10.1117/1.JBO.19.6.066003 24898603

[r43] Holmer A. et al., “Hyperspectral imaging in perfusion and wound diagnostics-methods and algorithms for the determination of tissue parameters,” Biomedizinische Technik 63, 587–594 (2018). 10.1515/bmt-2017-0155 30028724

[44] Bosschaart N. et al., “A literature review and novel theoretical approach on the optical properties of whole blood,” Lasers Med. Sci. 29(2), 453–479 (2013). LMSCEZ 1435-604X 10.1007/s10103-013-1446-7 PMC3953607 24122065

[45] Croce A. C. Bottiroli G., “Autofluorescence spectroscopy and imaging: a tool for biomedical research and diagnosis,” Eur. J. Histochem. 58, 2461 (2014). EJHIE2 0391-7258 10.4081/ejh.2014.2461 25578980 PMC4289852

[r46] Urban B. E. Subhash H. M., “Multimodal hyperspectral fluorescence and spatial frequency domain imaging for tissue health diagnostics of the oral cavity,” Biomed. Opt. Express 12(11), 6954–6968 (2021). BOEICL 2156-7085 10.1364/BOE.439663 34858691 PMC8606135

[47] Bjorgan A. Pukstad B. S. Randeberg L. L., “Hyperspectral characterization of re-epithelialization in an in vitro wound model,” J. Biophotonics 13, e202000108 (2020). JBOIBX 1864-063X 10.1002/jbio.202000108 32558341

[48] Gaffin D. D. et al., “Scorpion fluorescence and reaction to light,” Anim. Behav. 83, 429–436 (2012). ANBEA8 0003-3472 10.1016/j.anbehav.2011.11.014

