# Supplementary material for: Python-controlled multimodal UV-VIS hyperspectral imaging system
Source: J Biomed Opt. 2026 Sep 15;31(9):096003. doi: 10.1117/1.JBO.31.9.096003 (PMC13577617; doi:10.1117/1.JBO.31.9.096003)
Supplement: Supplementary file 1 [file JBO_031_096003_SD001.pdf]

Table S1: HySAF exposure times for blood sample imaging

Wavelengths (nm)	Exposure Time (ms)
440-480	200
490-500	100
510-520	70
530-540	40
550-560	30
570-590	18
600-620	12
630-650	8
660-680	5
690-730	4

Table S2: HySAF exposure times for nanoparticle imaging

Wavelengths (nm)	Exposure Time (ms)
440-480	400
490-520	150
530-550	80
560-580	40
590-630	20
640-690	10
700-730	7

Table S3: HySAF representative exposure times for white light imaging of rat wounds. A complete set of all exposure times is included in the metadata files for the rat hyperspectral images uploaded to HySAF's GitHub repository.

Wavelengths (nm)	Exposure Time (ms)
440	725
450	675
460	635
470	596
480	469
490	383
500	311
510	236
520	179
530	141
540	106
550	87
560	74
570	64
580	52
590	44
600	37
610	32
620	27
630	24
640	20
650	18
660	16
670	13
680	12
690	11
700	10
710	9
720	9
730	9

Table S4: HySAF representative exposure times for 385 nm autofluorescence imaging of rat wounds

Wavelengths (nm)	Exposure Time (ms)
420	1800
430	1100
440	600
450-520	400
530-610	500
620	900
630-700	1000

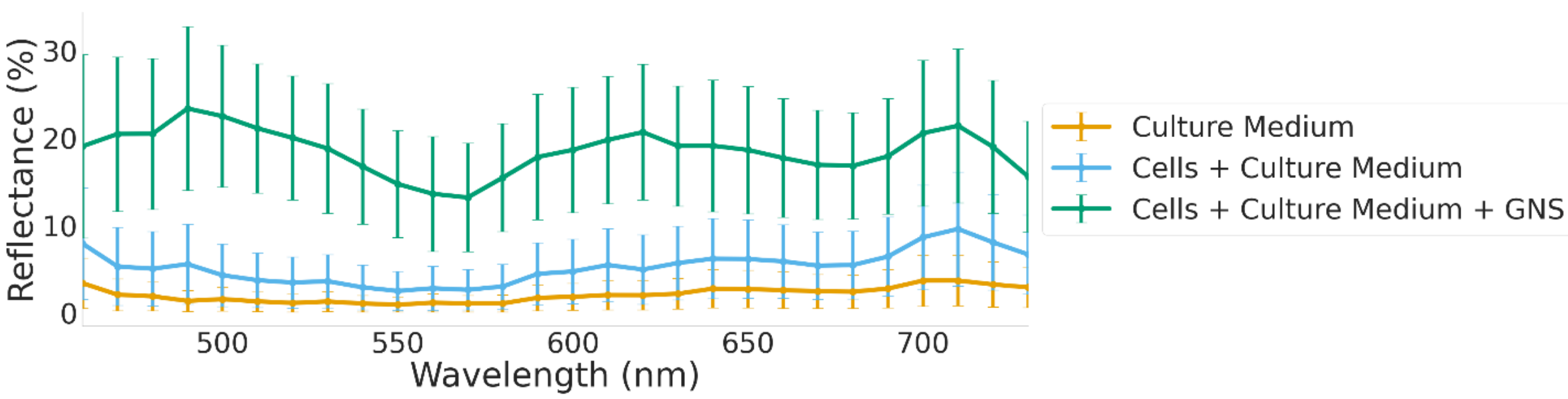

Figure S1: HySAF calculated reflectance and standard deviation values for cell culture medium alone [orange], THP-1 derived M0 macrophages in culture medium (uninteracted) [blue] and after 24 hours of interaction with bare GNS [green].
